# Rediscovery and redescription of the holotype of *Liolaemus lemniscatus* Gravenhorst, 1838 (Reptilia, Squamata, Liolaemidae)

**DOI:** 10.3897/zookeys.320.5372

**Published:** 2013-07-31

**Authors:** Bartosz Borczyk

**Affiliations:** 1 Department of Evolutionary Biology and Conservation of Vertebrates, University of Wroclaw, ul. Sienkiewicza 21, 50-335 Wrocław, Poland

**Keywords:** Chile, holotype, *Liolaemus*, redescription, Squamata

## Abstract

The presumed lost holotype of *Liolaemus lemniscatus* Gravenhorst, 1838 has been found at the Museum of Natural History of the University of Wrocław and identified by the individual pattern of head scales which matches Gravenhorst’s drawing. The first detailed description of this specimen is provided.

## Introduction

The former Zoologisches Museum Breslau, currently Natural History Museum of the University of Wroclaw (UWZM), housed a large collection of amphibians and reptiles including many type specimens. Unfortunately, many have been lost or are presumed to have been lost towards the end of World War II when the Museum building together approximately 75% of the city of Wrocław (then turned into Festung Breslau) were destroyed. Large parts of the collection catalogue went up in flames (Wiktor 1997). In the 50s of the 20^th^ century most of the specimens were placed in new jars and/or the preserving fluids were replaced. Unfortunately, in many cases the original labels were replaced, which may have occasionally led to some loss of information (see Wiktor 1997). The political and economic situation together with inadequate curation led to the further degradation of the collection. Regarding the Gravenhorst collection, the previous Museum’s Director Prof. Andrzej Wiktor stated that it had been lost except for a few insect specimens (an unpublished letter to Alain Dubois, see Dubois and Ohler 2000: 13). However, recent efforts to overhaul and ultimately to rebuild the collection yielded the holotype of *Liolaemus lemniscatus* Gravenhorst, 1838.

Currently, the genus *Liolaemus* (family Liolaemidae) comprises more than 230 species ranging from southern Argentina to northern Peru. There are numerous recent studies of the systematics, phylogeny and evolution of this group (e.g. Espinoza 1999, Etheridge 2000, Lobo and Espinoza 2004, Lobo 2001 and Abdala 2007, Lobo et al. 2012, Quinteros 2012).

*Liolaemus lemniscatus* was briefly described by J.L.C. Gravenhorst in 1838: “The dorsal scales form 25 rows and the belly scales 33 rows. Forehead with six pairs of consecutive enlarged scales. Drawing: Above warts light brown, with a black spot above the upper arm and on the sides with two long white lines and two rows of black and brown spots” (Gravenhorst 1838: 731). Next, a detailed redescription of this rediscovered holotype specimen is provided.

## Methods

The measurements and scale counts follow the description and terminology in Lobo (2001) and Abdala (2007) whereas the color pattern description follows the terminology in Lobo and Espinoza (1999). All measurements have been made using a digital caliper to the nearest 0.01 mm with the aid of a dissecting microscope.

## Results and redescription

**Figure 1.**
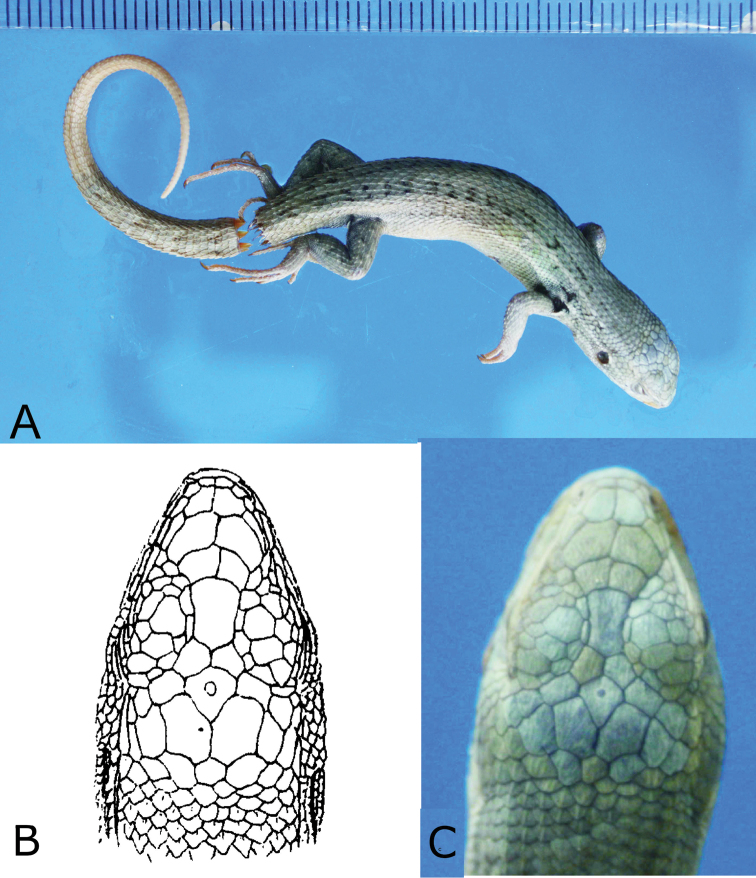
Holotype of *Liolaemus lemniscatus* Gravenhorst, 1838. **A** dorsolateral view **B** original drawing of the holotype (reprinted from Gravenhorst 1838) **C** dorsal view on head.

The specimen has been identified as the holotype on two grounds. First, there is a “n. spec. (?)” annotation on the label that remains on the jar. Inside the jar there is another label that was made in 1951 to replace the original one, and it holds only the species name with its authority. The species name is misspelled as “*Liolaemus lemnisodatus*” and the question mark suggests that the author of the label was not sure whether it really is the holotype of *Liolaemus lemniscatus*. Second, on comparison with the drawing of the holotype’s head in Gravenhorst (1838: table LIV, fig. 12; reprinted in Fig. 1C herein), there is an exact match in the pattern and shapes of the dorsal head scales between the specimen and Gravenhorst’s drawing. It is known that the variation in head scales size and shape permits individual recognition of lizards and snakes in the field (e.g., Borczyk 2000). It is thus reasonable to conclude that the specimen is indeed the one J. L.C. Gravenhorst used to describe *Liolaemus lemniscatus*.

The specimen is in a good condition. The only damage is a small hole near the left mouth corner for the lost label string (see introduction) and the separate tail stored together with the specimen. Also, the right anterior quarter of the belly bears a small wound that must have been incurred while it the individual was still alive as it seems to show the beginning of healing process.

*Holotype description*. Adult female UWZM Re 0027, collected in Valparaíso, Chile. The measurements (in mm) and scale counts are as follows: SVL 42.18; tail broken at 5.65 from the vent, the remaining part is 46.94 long with terminal 31.2 regenerated; head length (from anterior border of auditory meatus to tip of snout) 8.86; head width (at the anterior border of the auditory meatus) 6,98; head height 5.47; axilla-groin distance 20.46; tail width at the base 4.69; interorbital distance (between postorbital semicircles) 5.38; eye-auditory meatus distance 3.61; internarial distance 1.97; arm length 4.71; thigh length 6.01; shank length 7.77; foot length (from the ankle to the tip of the 4^th^ toe) 11.9. Subocular 3.3; preocular 0.68; rostral 0.62 length and 2.22 wide; mental 0.93 long and 2.3 wide; auditory meatus 1.93 high and 1.4 wide. Dorsal head scales slightly rough. Interparietal scale hexagonal, elongated posteriorly, surrounded by six scales and smaller than the parietals. Frontal scale pentagonal, elongated, with slightly concave lateral margins, anteriorly wider. Three enlarged supraoculars, the first being the largest. Four scales between the frontal and supercilliaries. Seven supercilliaries, strongly elongated. Canthal separated from nasal by one scale. The loreal region slightly concave. Nasal surrounded by seven scales, contacts the rostral. Three loreals, one in contact with subocular. Five supralabials, the 4^th^ and 5^th^ strongly elongated with posterior border of the 5^th^ scale oblique. Four infralabials. Four internasals. Orbit with 12 upper and 13 lower ciliares. Nineteen gulars between auditory openings; four scales in contact with 2^nd^ infralabial, the neck region scales smaller than dorsal scales; temporal and lateral neck scales keeled; lateral neck scales lanceolate, imbricate and carinate; 16 scales between posterior border of the auditory meatus and arm base; 40 dorsal scales between occiput and anterior surface of thighs; 80 scales between mental and cloaca. 42 scales around midbody. Dorsal scales are lanceolated, imbricated, keeled with mucron and lack interstitial granules. Dorsal scales are bigger than ventral scales. Number of infradigital lamellae on the 3^rd^ finger 14/15 (right/left); number of infradigital lamellae on the 4^th^ toe 20/21 (right/left).

*The color pattern as seen in the preservative*. Back and flanks grey-green. Vertebral field three scales wide at mid-length, vertebral line absent. Paravertebral field two scales wide, only slightly darker than vertebral field. Paravertebral markings almost black, tend to form a stripe along their dorsal margins anteriorly and to break up in two rows of spots toward the tail. Dorsolateral and ventrolateral stripes one-scale wide, creamy white. Lateral field darker than dorsolateral field. Belly creamy white. Barely visible stripes between jaws and throat.
